# Quetiapine-Induced Place Preference in Mice: Possible Dopaminergic Pathway

**DOI:** 10.3390/ph14020156

**Published:** 2021-02-14

**Authors:** Yusuf S. Althobaiti

**Affiliations:** 1 Department of Pharmacology and Toxicology, College of Pharmacy, Taif University, P.O. Box 11099, Taif 21944, Saudi Arabia; 2 College of Pharmacy, Addiction and Neuroscience Research Unit, Taif University, Taif 21944, Saudi Arabia; 3 Ministry of Interior, General Directorate of Narcotics Control, General Administration for Precursors and Laboratories, Riyadh 11543, Saudi Arabia

**Keywords:** quetiapine, reward, conditioned place preference, abuse

## Abstract

Quetiapine, an atypical antipsychotic, is effective in the management of schizophrenia, depression, and anxiety. Although quetiapine overdosage and misuse have been reported, its abuse potential has not been investigated in animals. In this study, the abuse potential of quetiapine was assessed based on the conditioned place preference (CPP) paradigm of drug addiction in a mouse model. First, mice received intraperitoneal injections of quetiapine (40, 80, or 120 mg/kg) every other day during the conditioning phase. In the second experiment, mice were pretreated with 0.03 mg/kg SKF-35866, a D1 receptor antagonist, before receiving saline or quetiapine (120 mg/kg) during the conditioning phase. No significant changes in time spent in the quetiapine-paired chamber were observed compared with time spent in the saline-paired chamber in mice treated with 40 or 80 mg/kg. In contrast, the preference to the quetiapine-paired chamber was significantly increased in mice treated with 120 mg/kg quetiapine, and this effect was blocked by SKF-35866 pretreatment. These results demonstrated, for the first time, the abuse potential of quetiapine in an animal model of drug addiction. Interestingly, this CPP-inducing effect was likely mediated by activating D1 receptors.

## 1. Introduction

Quetiapine, an atypical antipsychotic and derivative of dibenzothiazepine, is effective in the management of schizophrenia, bipolar disorder, and major depressive disorder, in combination with serotonin-norepinephrine reuptake inhibitors or selective serotonin reuptake inhibitors [1,2]. In addition, quetiapine has shown efficacy in the treatment of depression and anxiety as a monotherapy [3,4]. Notably, the effects of this drug are mediated by blocking various receptors, including serotonin (5-HT1A, 5-HT2A), dopamine (D2), adrenergic (α1, α2), and histamine (H1) receptors [5].

Some cases of quetiapine overdosage and misuse have been reported [6,7,8,9,10]. In a retrospective review of 3497 cases of atypical antipsychotic drugs (2118 of which were quetiapine and 1379 of which were other medications, such as risperidone, olanzapine, aripiprazole, clozapine, and ziprazidone), the intentional abuse of quetiapine was compared with that of other atypical antipsychotic drugs, and quetiapine was reported the most commonly abused atypical antipsychotic [11]. In another systematic, retrospective review of data gathered from a drug abuse warning network on quetiapine-related visits to the emergency department, a high number of visits related to the misuse and abuse of quetiapine was reported, suggesting high potential for abuse [12]. These cases of quetiapine abuse suggest that quetiapine could be a new abused agent. However, this drug is widely available and is not designated as a controlled substance, enabling abusers to obtain the drug easily without being arrested or punished.

In the central nervous system, several neurotransmitters can be affected by psychoactive drug intake [13]. For example, dopamine has strong effects on drug-seeking and reinforcing behaviors [14]. In fact, increased concentrations of dopamine in the brain regions involved in drug-seeking behaviors have been reported following exposure to several abuse-related drugs [15], and drug-seeking behaviors in response to several drugs can be prevented by blocking dopaminergic receptors [16,17,18]. In particular, dopaminergic D1 receptors have been reported to play major roles in the reward system in the brain [19,20,21,22,23]. However, motor functions have been consistently reported to be mediated through D2 receptors [24,25].

Preclinical studies of quetiapine abuse potential have yielded inconsistent results. In a conditioned place preference (CPP) animal study, 10, 20, or 40 mg/kg quetiapine did not show any effects on CPP when administered alone [26]. In another study that tested quetiapine abuse potential in rodents using CPP and self-administration animal models, the tested doses (0.1, 0.5, and 1 mg/kg) did not produce any significant increase in place preference scores. These CPP findings suggested that there was no abuse potential for these low doses. In self-administration experiments, animals engaged in frequent quetiapine self-administration, suggesting that this drug may have reinforcing effects [27]. However, the abuse potential of quetiapine remains unclear.

Therefore, in this study, the abuse potential of quetiapine was evaluated in a CPP model. Additionally, the possible mechanisms through which the drug may activate the reward system in the brain to promote abuse were also assessed.

## 2. Results

### 2.1. Experiment 1

**Figure 1:**
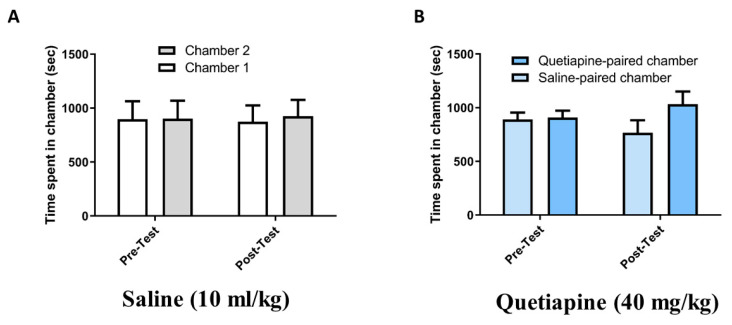
(**A**) Time spent in chamber 1 compared with chamber 2 during the pretest and post-test in the control group. (**B**) Time spent in the quetiapine-paired chamber compared with the saline-paired chamber during the pretest and post-test in the Quet-40 group (treated with quetiapine 40 mg/kg). Values are shown as means ± standard errors of the means.

In the control group treated with saline, two-way RM ANOVA revealed a nonsignificant main effect of phase (F (1, 7) = 1.000, *p* = 0.3506) or chamber (F (1, 7) = 0.1482, *p* = 0.9241) and a nonsignificant phase × chamber interaction (F (1, 7) = 0.2759, *p* = 0.8704; Figure 1A). In the Quet-40 group treated with quetiapine (40 mg/kg), two-way RM ANOVA revealed a nonsignificant main effect of phase (F (1, 9) = 1.040, *p* = 0.3345) or chamber (F (1, 9) = 0.7591, *p* = 0.4062) and a nonsignificant phase × chamber interaction (F (1, 9) = 1.811, *p* = 0.2114; Figure 1B).

**Figure 2:**
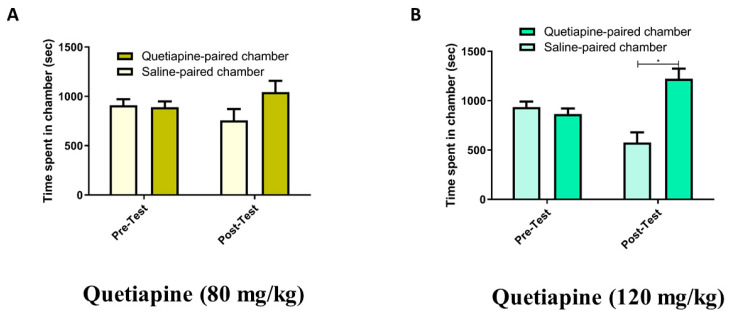
(**A**) Time spent in the quetiapine-paired chamber compared with the saline-paired chamber during pretest and post-test in the Quet-80 group (treated with quetiapine; 80 mg/kg). (**B**) Time spent in the quetiapine-paired chamber compared with the saline-paired chamber during pretest and post-test in the Quet-120 group (treated with quetiapine; 120 mg/kg). Values are shown as means ± standard errors of the means. *p < 0.05 compared with the saline-paired chamber.

In the Quet-80 group treated with quetiapine (80 mg/kg), two-way RM ANOVA revealed a nonsignificant main effect of phase (F (1, 7) = 1.000, *p* = 0.3506) or chamber (F (1, 7) = 0.9837, *p* = 0.3543) and a nonsignificant phase × chamber interaction (F (1, 7) = 1.517, *p* = 0.2578; Figure 2A). In the Quet-120 group treated with quetiapine (120 mg/kg), two-way RM ANOVA revealed a nonsignificant main effect of phase (F (1, 8) = 1.000, *p* = 0.3466), but a significant effect of chamber (F (1, 8) = 0.6.469, *p* = 0.0345) and a significant phase × chamber interaction (F (1, 8) = 9.112, *p* = 0.0166; Figure 2B). Newman–Keuls multiple comparisons tests showed that there was a significant increase in the time spent in the quetiapine-paired chamber compared with the saline-paired chamber during the post-test (*p* < 0.05). No significant changes were detected in the time spent in the quetiapine-paired chamber compared with the saline-paired chamber during the pretest. There were also no significant changes when comparing the time spent in any given chamber between the post-test and pretest.

### 2.2. Experiment 2

**Figure 3:**
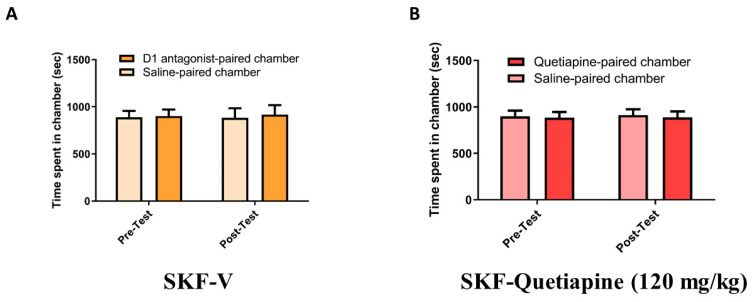
(**A**) Time spent in the D1 antagonist-paired chamber compared with the saline-paired chamber during pretest and post-test in the SKF-V group (treated with SKF followed by vehicle). (**B**) Time spent in the quetiapine-paired chamber compared with the saline-paired chamber during pretest and post-test in the SKF-Quet group (treated with SKF followed by quetiapine). Values are shown as means ± standard errors of the means.

In the SKF-V group treated with SKF (0.03 mg/kg) followed by saline, two-way RM ANOVA revealed a nonsignificant main effect of phase (F (1, 6) = 1.000, *p* = 0.3559) or chamber (F (1, 6) = 0.02992, *p* = 0.8684) and a nonsignificant phase × chamber interaction (F (1, 6) = 0.007901, *p* = 0.9321; Figure 3A). In the SKF-Quet group treated with SKF (0.03 mg/kg) followed by quetiapine (120 mg/kg), two-way RM ANOVA revealed a nonsignificant main effect of phase (F (1, 7) = 2.320, *p* = 0.1715) or chamber (F (1, 7) = 0.03032, *p* = 0.8667) and a nonsignificant phase × chamber interaction (F (1, 7) = 0.004477, *p* = 0.9485; Figure 3B).

## 3. Discussion

In this study, the ability of quetiapine to induce CPP in mice was discovered. The important roles of D1 receptors in this effect were also elucidated. The findings suggested that quetiapine activated the reward system in the brain, which could explain the increased place preference found in this study with the highest tested dose of 120 mg/kg. Notably, one preclinical study contradicted the current findings and concluded that quetiapine did not show any addictive potential because it did not affect place preference in the tested rats [26]. However, this previous study tested lower doses of quetiapine (10, 20, and 40 mg/kg) compared with the doses used in the current study (40, 80, and 120 mg/kg). Consistent with this previous study, CPP was not affected by 40 mg/kg quetiapine in the current study. Interestingly, when the dose was increased to 80 mg/kg, preference tended to increase, although the change was not significant. Furthermore, a significant preference for the quetiapine-paired chamber was found when the dose was increased to 120 mg/kg. This suggested that the drug had dose-dependent effects on the reward system in the brain. Importantly, the findings in this study were supported by several case reports of quetiapine abuse/misuse at higher doses. For example, a 34-year-old female subject with a history of substance use disorder crushed her pills, dissolved them in water, and injected herself with 600 mg quetiapine [28]. Similarly, several reports have described cases of patients with a history of polysubstance abuse who snorted [29] or received [30] high doses of quetiapine and experienced rush sensations and withdrawal symptoms. In addition, a retrospective study was conducted on cases of abuse/misuse of quetiapine from poison centers in the USA from 2005 to 2011. In total, 1948 cases met the criteria for misuse, defined as improper use of drugs, such as increasing the dose to enhance its effects, whereas 1168 cases met the criteria for abuse, i.e., the intentional use of the drug to get high, including recreational use; higher numbers were observed among adolescents [31].

Quetiapine has multiple street names, including “quell,” “Susie-Q,” “baby heroin”, and “Q-ball” [32,33,34,35]. Thus, the drug is a known, abuse-related drug among addicts, further supporting the current findings on CPP induction in a mouse model of drug addiction. Some previous studies have been conducted in animals to elucidate the effects of quetiapine and determine whether the drug induced addiction. In a study of the potential dependence of quetiapine in mice, researchers used the same CPP method, but lower doses (5, 7, and 10 mg/kg) of quetiapine and found a dose-dependent effect; however, the result was not statistically significant. In self-administration experiments, animals engaged in frequent self-administration of quetiapine, suggesting that this drug may have reinforcing effects [27].

Because the drug can function by blocking various receptors, including serotonin (5-HT1A, 5-HT2A) receptors and dopamine (D2) receptors [36,37], quetiapine can increase the release of dopamine [38]. Accordingly, quetiapine abuse may be driven by D1 receptor activation, which is well known to activate the reward system in the brain and lead to addiction [39]. Although the exact mechanism through which quetiapine can induce reward is not known, several well-known, abuse-related drugs have been shown to induce drug-seeking behavior through the modulation of dopamine [40]; the same mechanism may be involved in quetiapine abuse. Rewarding and reinforcing behaviors have been shown to be induced by dopamine release in the nucleus accumbens and D1 receptor activation [41,42]. Interestingly, SKF-83566 has been shown to interfere with dopamine release induced by cocaine and block hyperlocomotion caused by amphetamine exposure [43,44]. In this study, the effects of blocking D1 receptors were tested by pretreating mice with a D1 antagonist, SKF-35866, before the administration of the dose of quetiapine that induced CPP (120 mg/kg). This pretreatment with SKF-35866 completely blocked CPP to quetiapine, suggesting that D1 receptors play important roles in activating the reward system of the brain in response to quetiapine.

One limitation of this study is that dopamine release and its concentration in the key brain regions of the reward pathway were not assessed following exposure to quetiapine. Further studies are needed to explore the neurobiological changes induced by quetiapine.

## 4. Materials and Methods

### 4.1. Animals

Male BALB/c mice (King Fahd Medical Research Center, Jeddah, Saudi Arabia), weighing 20–30 g at the beginning of the study, were housed in standard plastic tubes with controlled humidity (30%) and temperature (21 °C) on a 12:12 light–dark photoperiod, and were allowed to habituate for 7 d before experiments. Mice were provided food and water ad libitum throughout the experiment. All experiments were performed during the light cycle. In accordance with the Institutional Animal Care and Use Committee of the National Institutes of Health guidelines, the experimental procedures of the animal study were approved by the Research Ethics Committee at Taif University.

### 4.2. Drugs

Quetiapine was a donation from Riyadh Pharma (Riyadh, Saudi Arabi). SKF-83566 was obtained from Tocris Bioscience (Ellisville, MO, USA). Saline solution vehicle (0.9% NaCl) was used to reconstitute all drugs used in this study.

### 4.3. Experimental Design

Experiment 1: Animals were randomly separated into four groups: control group (*n* = 8), administered vehicle (10 mL/kg, intraperitoneal (i.p.) injection) for 8 d; Quet-40 group (*n* = 8), administered quetiapine (40 mg/kg, i.p. injection four times) and vehicle (10 mL/kg, i.p. injection four times) for 8 d during the conditioning phase; Quet-80 group (*n* = 8), administered quetiapine (80 mg/kg, i.p. injection four times) and vehicle (10 mL/kg, i.p. injection four times) for 8 d during the conditioning phase; and Quet-120 group (*n* = 8), administered quetiapine (120 mg/kg, i.p. injection four times) and vehicle (10 mL/kg, i.p. injection four times) for 8 d during the conditioning phase.

Experiment 2: Animals were randomly assigned to two groups: SKF-V group (*n* = 7), administered SKF-83566 (0.03 mg/kg, i.p. injection) 30 min before vehicle (10 mL/kg, i.p. injection eight times) for 8 d during the conditioning phase; and SKF-Quet group (*n* = 8), administered SKF-83566 (0.03 mg/kg, i.p. injection) 30 min before quetiapine (120 mg/kg, i.p. injection four times) for 8 d during the conditioning phase.

### 4.4. CPP Paradigm

The CPP apparatus was made of acrylic and consisted of two conditioning chambers, which were identical in size and distinguished by both tactile and visual cues, as described previously [45]. In the habituation phase (days 1–3), each mouse was placed in the start box with the door closed for 3 min. Then, the door was opened, and the mouse had free access to both chambers for 30 min. On day 3, mouse movement in the two chambers was recorded (pretest) and the time spent in each chamber was calculated using the ANY-maze software (Stoelting, USA). An unbiased CPP design was followed and, to eliminate possible bias to any chamber, mice spending >67% of the total time in any one chamber were excluded from the study [45,46,47]. Moreover, half of the animals were randomly assigned to receive quetiapine in chamber 1 and the remaining half received it in chamber 2.

**Figure 4:**
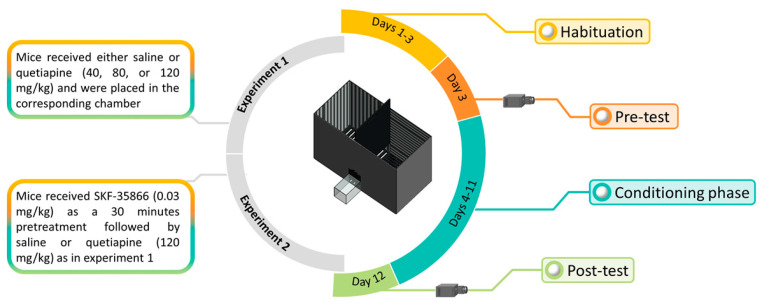
Experimental schedule of the conditioned place preference (CPP) experiments.

In the conditioning phase (days 4–11), each mouse received the selected dose of quetiapine according to the assigned group and was confined, immediately after the injection, into the designated chamber for 30 min. The next day, each animal received the vehicle dose (10 mL/kg) and was confined into the opposite chamber for 30 min. On day 12, mouse movement in the two chambers was recorded (post-test) and the time spent in each chamber was calculated using the ANY-maze system. In experiment 2, the same process was performed during the conditioning phase, except that animals received a 30-min pretreatment with SKF-83566 before the administration of saline or quetiapine (Figure 4).

### 4.5. Statistical analysis

Two-way repeated measures (RM) analysis of variance (ANOVA; phase × chamber) was used to analyze the time spent in the conditioning chambers. Newman–Keuls comparisons were applied when significant main effects or interactions were identified. All data were statistically analyzed using GraphPad Prism, with a significance level of 0.05.

## 5. Conclusions

The findings of this study showed, for the first time, that quetiapine could cause drug-related effects in CPP experiments in mice. The results also showed that this CPP-inducing effect was likely mediated by activating D1 receptors, as is addiction. Based on this important evidence, the use of this drug should be restricted. Healthcare providers should be aware of the important potentially-addictive effects of this drug and plan prescriptions accordingly, especially in patients with a history of substance abuse disorder. The concerned authorities may consider these findings when establishing guidelines for the restricted use of quetiapine to decrease its abuse potential.

## Data Availability

Available upon reasonable request.
